# Michigan State Dental Society

**Published:** 1887-06

**Authors:** J. A. Watling


### PROCEEDINGS OF SOCIETIES.

### Michigan State Dental Society.

### WHAT SHALL WE DO WITH BADLY DECAYED FIRST MOLAR TEETH AT AN EARLY PERIOD IN LIFE.

BY DR. J. A. WATLING.

Shall they be filled or taken out with the view of allowing the second or third molar teeth to crowd forward and fill up the places, and at what period is it proper to remove these teeth? It is a very common thing for people to come to me with teeth that have been filled at eight, ten, or twelve years; they were filled with amalgam and had gone to pieces in four, five, or six years. It is a question whether these teeth had better have been taken out or filled. It is a matter that requires much judgment. I have here a model where the teeth have been taken out with the view of taking models. The patient was eleven years of age. They had been filled with amalgam. Now I have removed these teeth and expect in two years time to have the second molar occupy the position that the first molar occupied. I claim that the upper teeth can be extracted at a much earlier period than the lower, with the view of their filling up. At the age of seventeen the first molar may often be taken out with good results. My own mouth proves that. At the present time the second molars occupy the place of the first, and the third molars are good. Now, in the absence of my models and notes I cannot say much more.

What I have said is more a matter of eliciting discussion. I would like the views of others, and get their experience in this matter. I would not defend the wholesale destruction of these teeth; where there is a probability of saving them, fill them by all means. If they are to be lost at what time are they to be sacrificed, at the age of eleven or twelve, or at the age of twenty or thirty?

DISCUSSION.

*Dr. Field.*—This case that Dr. Watling has shown us, seems to me, to be hardly a fair case. I should say that these teeth had been teeth in which there had been sickness at some time during childhood, where there had been want of nutrition. Now, it might be a question as to whether such teeth would be proper for us to attempt to fill. If Dr. Watling takes the ground that we should extract the teeth when they begin to decay, I take issue with him at once. I should fill them temporarily with phosphate and wait for them to calcify. The extracting of these teeth I have found to cause the pushing forward of the molars that are to fill the space that makes a bad shape between the cuspid and the twelve year molar. It is not only a matter of time when these teeth should be extracted, but a matter of their structure. I should save every tooth that I could for a child. Save them with temporary fillings. I would take a dead tooth out after the child was eight, ten, or twelve years old.

*Dr. Long.*—At what age would you prefer to extract these teeth?

*Dr. Field.*—That would depend very much upon the condition of the teeth.

*Dr. Jackson.*—I think in the extracting of these teeth we ought to take into consideration each and every particular case. If you take them out before the jaw is developed it interferes very much with the development of the lower jaw. Then we must take into consideration the chalky condition of the teeth of the patient. If there is no softening down along the margin of the gums, I should try filling the teeth. It is frequently the case that between eighteen and twenty the teeth that a few years before would seem impossible to save may have changed so that it is easily done now.

*Dr. Taft.*—I do not suppose that any rule will be applicable to every practitioner. All are not alike in their apprehension of the condition of things. Some men manipulate well, while others poorly. Different individuals see things differently. You hold an object before a number of men and ask them " What do you see?" Each will give different expressions, each show a difference of apprehension. This applies to the treatment of these teeth as well. A man who has made a study of such teeth is better able to instruct and give the results that would come from any course of treatment. It is well for every man to study himself in this particular. Men who are working in the dark should be cautious in the methods of their proceedure. If all men were alike in ability, there would be other things that would modify their proceedure. If all teeth were alike then it would be easy to arrive at a uniform method of treatment. Sometimes it is found that teeth can be treated with results far beyond expectation. I remember more than thirty years ago of filling teeth with tin, thinking that the teeth would soon be lost. These teeth improved in texture, and the fillings remained in them more than twenty years. They now have gold fillings. If the first permanent molar has decayed, and there is a probability that the jaws will expand so as to give place to the third molar, then save the first permanent molar. It is generally a risk in removing these teeth. In many instances the other teeth will come together, not always. One individual takes one view only, he says unhesitatingly, that all first molar teeth should be taken out. Another man says that all the teeth should be saved. So these varieties account for the difference of opinion of the participators in this discussion. An elective course in respect to the treatment of these teeth is the best. We should study the cases where the teeth are liable to change position in the mouth. The loss of a tooth for a year or two is not a serious objection. Another point is the position the teeth may take after they do change position. These teeth will sometimes approximate one another and not come together at all. This we should take into account, as we cannot always anticipate results. In case regulating is to be done then remove the teeth. On the other hand, where the teeth are very weak, the first molar should, by all means, be taken out. The first bicuspid should very seldom be taken out. I think we need to get a more intelligent apprehension of this subject.

*Dr. Long.*—One thing I would like to know. In many cases the third molar never appears, what would be done in this case?

*Dr. Watling.*—I would not fill these teeth unless I was quite certain of success. Persons come in here where dentists had no hopes of saving these teeth more than five years.

They come when the space between them would not fill up. If these teeth are out before the lower jaw is fully grown, in nine cases out of ten, they will close up. You may carry it to the age of twelve or fourteen, and the space will fill up. I would save these teeth if there is a prospect of their being good.

DISCUSSION UPON DR. LONG'S PAPER.

*Dr. Taft* —I have only a question to ask. I understand the author of the paper to say that nitrous oxide would not support life. How does he explain the fact that persons can inhale nitrous oxide alone from ten to thirty minutes and be as much alive at the end of that time as at the beginning?

*Dr. Long.*—Mr. President :—I simply generalized that. I did not suppose that it would be taken that they could not live ten or thirty minutes, but they could not live two or three days. Gas can be given with perfect safety. I know of a case where a man took five hundred gallons in forty-eight hours. He said his head was dizzy, and he was drunk for four days after.

*Dr. Land.*—I have a faint recollection of a case of experiment made in Paris with nitrous oxide. I believe it was said that if a person could be fed he could be made to live by changing the specific gravity.

*Dr. Metcalf.*—I would like to inquire of Dr. Long if there is not a distracted period, and disturbed condition of the mind, and if there is, if there is not an excited condition of the pulse?

*Dr. Long*—If there is an excited condition of the pulse I do not believe that it is due to the anæsthetic. I think that this excitement is where many have been mislead. I have said so much to-day, but there is one more suggestion I would like to make, that those who manufacture their own gas should not keep it over water. It takes double and sometimes three times the amount. Collect the gas in a gasometer and then with an air pump condense or liquify it, then have a reservoir made and force it into this and keep it there until ready for use. I think there is probably some waste, but it answers the purpose and keeps it much better than over water.

*Dr. Taft.*—A question as to the stability of this compound. How does it compare with the atmosphere? The change of the nitrous oxide is probably due to the decomposition of the gas, and the absorption of the oxygen by the water. I would like Dr. Vaughn's opinion on this matter.

*Dr. Vaughn.*—I have listened to the discussion on this subject with a great deal of interest. In the first place, nitrous oxide when taken into the blood forms a chemical compound with the coloring matter of the blood. When we breathe nitrous oxide gas it forms nitrous oxide hemoglobine, both of these compounds are very loose and unstable. Carbon monoxide is given off from coal stoves. This forms a very stable compound with the hemoglobine of the blood. We had an illustration in Detroit a short time ago. Nitrous oxide outside of the blood is a very unstable compound.

*Dr. Taft.*—A question that is not quite clear to me. Whether the decomposition did not take place before it was taken into the blood at all and the different manifestations being due to the greater amount taken in, or whether this is due to the different conditions of the oxygen or the taking in of both oxygen and nitrogen.

*Dr. Vaughn.*—I don't think that can be so for the effects on hemoglobine of oxygen and condensed oxygen are very different.

*Dr. Long.*—I would like to ask for information from Dr. Vaughn. I understand that nitrous oxide acts as a hemoglobine. I would like to ask whether hemoglobine would be able to use up all the oxygen, or whether part of it would be eliminated as from nitrous oxide?

*Dr. Vaughn.*—I don't know that I am prepared to answer that question. Of course the blood can take up only a limited amount of nitrous oxide. It is a question whether you take a man who is breathing ordinarily to what extent the nitrous oxygen would take the place of oxygen.

*Dr. C. R. Butler.*—Mr. President: I appreciate this compliment, and cannot forbear saying a few words just at this point. I am pleased to do so in the presence of these who will come in as our confreres in practice. I would much rather speak to them than to those venerable members of the profession, because they are reputed as wise, and these younger ones wish to be wise and that is laudable. Now to these young friends I would say, about all that is necessary is that you are really in earnest, for the remarks that Professor Taft has made that the physician and the dentist should serve humanity for the love, not the money, are true. If you serve for the love, the money will come as one of the consequences. The idea that we must strive to make a living is all right. I have not the least particle of sympathy for a lazy man. There is no room in this world for such characters. The Great Architect of the universe has given us the grandest example of industry we could wish to have, and shall we pay Him such a compliment as to be lazy in the use of the powers that we have been blessed with? The fear that these dental and medical colleges shall turn out more doctors and dentists than needed, there is no cause for. There is no danger of this being done for more reasons than one. Statistics show us that not more than one in seven who come into our profession make success as professional men. I trust that there will be more than one in seven that go out of this institution that will make a success as dentists, for there is room for all such, and just so sure as you are in earnest, are faithful and honest, you will be accorded all that is your due as professional men. It will be your own fault if you do not arrive at that position in the profession to which you undoubtedly aspire. I don't question that you have had excellent training, for I know something of the long preparation and earnest effort of some of those under whose teaching you have been sitting, and

I can say that the opportunities you are having here are far better than I had at your time of life, and yet I may be allowed to say it Mr. President, ladies, and gentlemen, that it is well known that by earnest effort, if nothing else, I have attained to something like a respectable position in the profession. These things do not come by magic, neither do they come by forming friends, but they come from earnest faithful friends, and you can only make such by being earnest and honest with those that come in hand.

A CASE.

The following case of an impacted third molar, inferior, was presented by Dr. M. W. Lau : the subject, a lady, Mrs. J. S., age 57. Six years ago she suffered great pain in the region of the inferior right third molar. She was advised by the physicians to have the first permanent molar removed, though it was not specially decayed. This was done but without giving the relief she sought. Then several other teeth were removed. The wound made by the removal of the second molar did not heal, and during the last year she has had much severe pain. I saw the case about two years ago, simply examined it, and was not able to arrive at a conclusion as to its character. She has been for some time under treatment in Detroit. There has never been a third inferior molar erupted.

At this point the lady was presented and took the chair. The case was examined by the members of the body, and after obtaining the history of the case, it was decided to be one of impacted third molar. Professor Watling was requested to make an effort for its removal, to which he consented. A free incision was made through the gum and contact made with the tooth. It was, however, well nigh covered with alveolus. This was cleared away, and the tooth more exposed. After quite a prolonged operation it was seized by a pair of forceps and removed. This was done without an anæsthetic, the patient suffering great pain, and when the operation was accomplished she was quite prostrated, but the indications were that she would have entire relief from the difficulty under which she had been so long suffering.

### Minutes of the Michigan State Dental Society.

The annual meeting of the Michigan State Dental Society occurred at Ann Arbor, March 22d to the 24th, inclusive. The morning and evening sessions were devoted to the reading of papers, and discussions, and the afternoon sessions to clinical work, excepting the last afternoon, which was devoted to both discussions and clinical work.

EVENING SESSION—MARCH 22, 1887.

The meeting was called to order at 7.30 p. m. by the President, Dr. Davis, with Dr. J. B. McGregor as Secretary.

The minutes of the previous meeting were read, and there being no objection, they were adopted as read.

The report of the University Visiting Committee was called for, but as they were not ready to report it was put over.

Opportunity to pay dues was given, and the Treasurer was authorized to take all such monies.

Dr. W. H. Dorrance entertained the Society with a talk on the subject of "Impressions." Topic was discussed by Drs. Harroun, Dorrance, Douglass, Land, and Taft

On motion the following hours for the meeting of the Association were adopted: From 9 a. m. to 12 m., from 2 p. m to 5 p. m., and from 7.30 p. m. to 9 p. m.

The time for the election of officers was fixed for Thursday at 11 o'cleck. Adjourned.

MORNING SESSION—SECOND DAY.

Meeting called to order by the President, Secretary Dr. J. B. McGregor at his post.

The Board of Censors reported Frederick M. Thompson as a candidate for admission to the Society. Rules were suspended, and Frederick M. Thompson was elected by acclamation.

Report of Board of Directors read, and accepted on motion.

Dr. C. H. Land read a paper on "Metallic Enamel."

Topic discussed by Drs. Moore, Land, Harroun, and Dorrance.

Dr. M. A. Long presented a paper on "Nitrous Oxide."

Adjourned.

EVENING SESSION—SECOND DAY.

Meeting called to order. Roll called by the Treasurer.

The Board of Censors reported the following names as candidates for membership in the Society: Drs. Wagals, W. W. Scott, Adelbert, C. J. Hand, and Miss Mosier. An affirmative vote being cast they became members by the payment of dues.

Dr. Vaughn, Prof. of Physiological and Pathological Chemistry in the University of Michigan, read a very interesting and instructive paper on "Salivary Analysis." Upon motion of Dr. Land the Society extended a vote of thanks to Dr. Vaughn for his highly interesting paper.

Topic discussed by Drs. Taft, Vaughn, Metcalf, Jackson, Robinson, and Land.

The discussion of Dr. Long's paper was participated in by Drs. Taft, Long, Metcalf, Land, and Vaughn.

Dr. G. C. Corbin read a paper on the subject "Should Physicians Extract Teeth?"

Topic discussed by Drs. Taft and Robinson.

Dr. C. R. Butler, of Cleveland, was called upon for remarks and responded in a very appropriate and complimentary manner.

Dr. Watling moved that Dr. Vaughn be requested to present his paper to the Society for publication in their proceedings. Dr. Vaughn very cordially complied with the request.

On motion the meeting was adjourned to reconvene at 9 o'clock Thursday morning.

MORNING SESSION—THIRD DAY.

Meeting called to order with President Davis in the chair.

Dr. Ricks was reinstated as a member of the Society.

The Board of Censors proposed the names of Drs. L. Gish and H. H. Dawley, who, upon the vote of the Society and payment of dues, became members.

Dr. Taft brought Dr. Stowell's new work "The Microscopic Structure of the Human Tooth" before the Society, and recommended it as one that should be in the hands of every dentist.

Dr. Watling gave a talk upon the subject "What Shall we do with Partly Decayed First Molar Teeth?"

Topic discussed by Drs. Field, Long, Watling, Jackson, and Taft.

The Society proceeded to the election of officers, which resulted in the choice of the following: President, Dr. J. A. Robinson; First Vice-President, Dr. G. C. Corbin; Second Vice-President, Dr. Cornes, and Treasurer, Dr. H. K. Lathrop.

Ann Arbor was designated as the place of the next meeting of the Society, to be held on the Tuesday before the last Saturday of March, 1888. Adjourned.

AFTERNOON SESSION—THIRD DAY.

Dr. C. H. Harroun read a paper on "Hare Lip and Cleft Palate," a case in practice.

The following was offered by Dr. Classon: Resolved, that it is the sense of this Society to assist the Board of Examiners in carrying on the work of the Board, and to aid in suppressing the illegal practice of Dentistry in this State. Therefore, be it resolved, that the Secretary of the Board be requested to issue a call on every registered dentist in this State for a contribution of one dollar to aid in this work.

After discussion the resolution was adopted.

Drs. Sweetman and M. B. Dennis were reported favorably by the Board of Censors, and after complying with the rules of the Society, became members.

Dr. C. R. Butler was made an honorary member of the Society.

The following committees were appointed:

Executive Committee, { J. A. Watling, J. Lathrop, C. D. Case.

Publishing Committee, { A. M. Long, G. B. Watkins, J. B. McGregor.

University Visiting Committee, J. B. McGregor in place of E. C. Moore.

Delegates to the American Dental Association, { J. L. Gish, C. I. Case, E. G. Douglass, G. E. Saunders, W. H. Miller.

It was moved and carried that the Society adjourn to meet the Tuesday before the last Saturday in March, 1888.

Society adjourned.

